# Investigation of the morphometry of the pre-lacrimal recess of the maxillary sinus for the pre-lacrimal approach of the maxillary sinus and paramedian skull base. A computed-tomography study

**DOI:** 10.25122/jml-2022-0112

**Published:** 2022-06

**Authors:** Florin Lupu, Cătălina-Petruța Iliuță, Ioan Alexandru Bulescu, Mihaly Enyedi, Daniela-Elena Gheoca Mutu, Octavian Enciu, Florin Mihail Filipoiu

**Affiliations:** 1 Department of Morphology, Doctoral School, University of Medicine and Pharmacy Carol Davila, Bucharest, Romania; 2 Department of Morphology, University of Medicine and Pharmacy Carol Davila, Bucharest, Romania; 3 Surgery Department, University of Medicine and Pharmacy Carol Davila, Bucharest, Romania

**Keywords:** maxillary sinus, pre-lacrimal recess, nasolacrimal duct, PLR – Pre-lacrimal recess, PLRA – Pre-lacrimal recess approach

## Abstract

The pre-lacrimal recess approach is modernly used for lesions of the anterior maxillary wall and for reaching paramedian cranial base regions. In this computed-tomography study, we assessed the pre-lacrimal recess types as well as the angles between the anterior and medial maxillary walls and between the anterior maxillary wall and the lateral margin of the nasolacrimal canal to show the feasibility of the pre-lacrimal recess approach in reaching lesions of the infratemporal and pterygopalatine fossae, using 30 computed-tomography studies (60 sides). A type I pre-lacrimal recess was identified in 22 cases (35%), type II was identified in 31 cases (53.30%), and type III in 7 cases (11.66%). We found that angle 1 (the angle between the anterior maxillary wall and the medial maxillary wall) had a mean value of 80.8° (minimum 75.5°, maximum 85.8°), while angle 2 (the angle between the anterior maxillary wall and the lateral margin of the nasolacrimal canal) had a mean value of 59.1° (minimum 57.6°, maximum 60.1°). We consider the pre-lacrimal recess approach a very good option for the anterior maxillary wall, the alveolar recess, and in reaching the infratemporal fossa and lateral part of the pterygopalatine fossa. In cases where direct visualization of the medial part of the pterygopalatine fossa is needed, the pre-lacrimal recess approach could not be the perfect option.

## INTRODUCTION

Endoscopic sinus surgery is nowadays the preferred technique for most inflammatory and benign lesions of the nose and paranasal sinuses since they are minimally invasive and functional. Several approaches have been described for lesions of the maxillary sinus. One of them is the pre-lacrimal recess approach (PLRA) of the maxillary sinus, especially used for the anterior and inferior walls of the sinus since they can be difficult to reach, even for experienced surgeons [1, 2]. This approach can also be used for reaching lesions in the paramedian middle cranial base, such as the infratemporal and pterygopalatine fossa [3, 4]. The pre-lacrimal recess is a concave region in the anterior and superior part of the maxillary sinus, anterior to the lacrimal passage on the medial maxillary wall [5]. The distance between the anterior wall of the maxillary sinus and the nasolacrimal duct was classified by Simmen et al. into three types: type I with the distance between 0–3 mm; type II between 3–7 mm, and type III, in which the distance is larger than 7 mm [1]. In this study, the authors concluded that a safe PLRA is only possible in type 3 (>7mm), so only in 12.5% of maxillary sinuses. Even in this case, a study by Luat Vien Tran et al. concluded that a modified technique of PLRA is safe and effective for the management of inverted papilloma even in type I lacrimal recess configuration [6]. Even though the PLRA is an effective approach for reaching and resolving lesions in the anterior part of the maxillary sinus, the question remains if PLRA can always be used for reaching the paramedian regions of the cranial base without injury to the nasolacrimal duct. This study was conducted to assess the feasibility of this approach related to the pathway of the nasolacrimal duct through the maxillary sinus.

## MATERIAL AND METHODS

**Figure 1:**
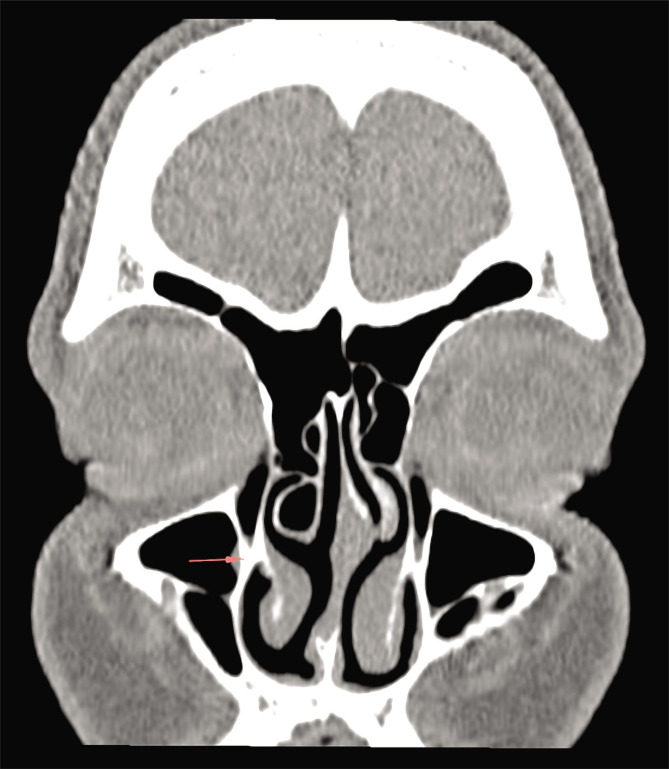
Coronal plane CT scan showing the insertion of the inferior turbinate on the frontal process of the maxillary bone.

**Figure 2:**
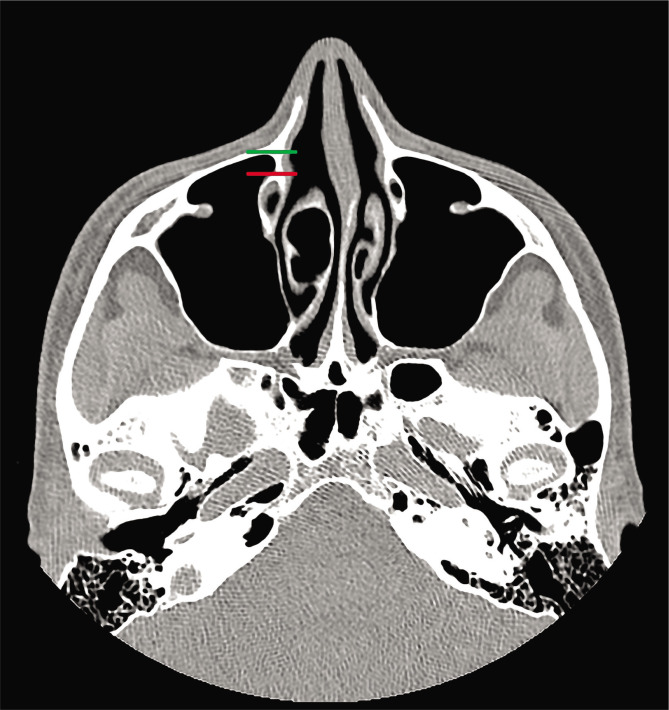
Transverse plane CT scan showing the lines used for measurements between the anterior maxillary wall (green line) and anterior margin of the nasolacrimal duct (red line).

**Figure 3:**
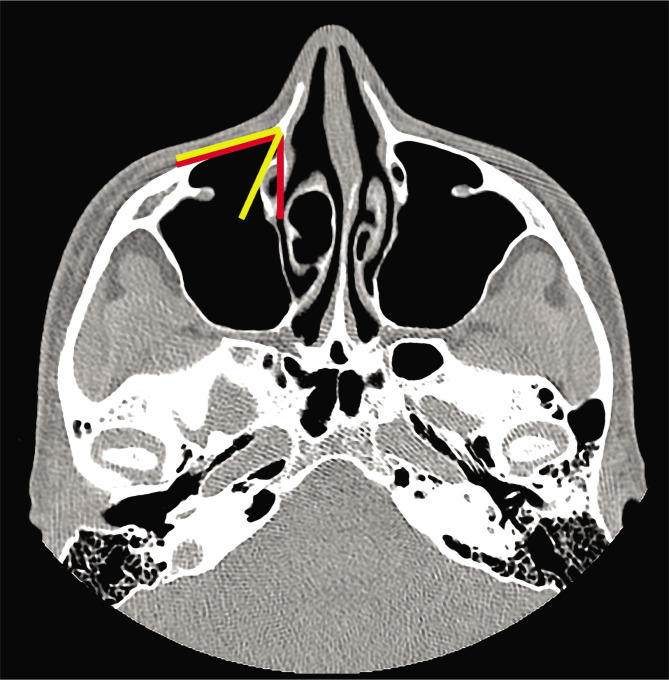
Transverse plane CT scan showing the measurements of the angles: the angle between the anterior and medial maxillary walls (angle 1) – red lines, and the angle between the anterior maxillary wall and the lateral margin of the nasolacrimal duct (angle 2) – yellow lines.

This is an anatomical imaging study in which we evaluated the area of the pre-lacrimal recess of the maxillary sinus using 30 computed-tomography studies of the nose and paranasal sinuses from our department's collection. We included in the study group only high-quality computed-tomographic images (at most 1.25 mm slices) from adult individuals with no sign of previous sinus surgery or destructive lesions of the nose and paranasal sinuses. We assessed the space available for surgical access for the maxillary sinus and the pterygopalatine and infratemporal fossae. The measurements were performed after identifying the insertion of the inferior turbinate onto the frontal process of the maxillary bone in the coronal plane (Figure 1) and transition into the transverse plane. All measurements were made in the same transverse plane for both right and left sides. The distance between the anterior wall of the maxillary sinus and the anterior margin of the nasolacrimal duct was measured (Figure 2), and the cases were divided into three types, according to the classification provided by Simmen et al. [1]. After classification, only cases classified as type II and III were chosen, and for those, we measured the angle of the opening between the anterior maxillary wall and the medial maxillary wall, as well as the angle between the anterior maxillary wall and the plane passing tangent to the lateral margin of the nasolacrimal duct (Figure 3).

## RESULTS

For our entire study series (60 maxillary sinuses), we identified a type I PLR in 22 cases (35%), a type II PLR in 31 cases (53.30%), and type III in 7 cases (11.66%). In the 38 cases classified as type II and type III PLR, we measured the angles between the anterior maxillary wall and medial maxillary wall (angle 1) and the angle between the anterior maxillary wall and the plane passing tangent to the lateral margin of the nasolacrimal duct (angle 2).

Angle 1 had a mean value of 80.8° (minimum 75.5°, maximum 85.8°), while angle 2 had a mean value of 59.1° (minimum 57.6°, maximum 60.1°), with no significant differences between right and left sides. The difference between angle 1 and angle 2 was determined by the width of the nasolacrimal duct in the plane of the section where we performed the measurements.

## DISCUSSION

The maxillary sinus has a complex and variable anatomy, with some hidden and hard-to-reach areas, especially close to the anterior wall and alveolar recess. These regions sometimes pose problems for visualization and access, even for experienced surgeons [1, 2, 7]. Several approaches have been proposed for resolving issues with hard-to-reach areas of the maxillary sinus (canine fossa approach, midfacial degloving, medial maxillectomy etc). However, some of these approaches are associated with increased postoperative morbidity [1, 8]. The PLRA to the maxillary sinus permits visualization and instrumentation of the most difficult areas within the maxillary sinus with favorable results and very low morbidity [9]. Some authors have also proposed that the PLRA can be used safely in surgery for removing tumors of the pterygopalatine and infratemporal fossae [3, 4, 10].

Even though good results were obtained, the PLRA is not always a viable option. According to Simmen et al. [1], the possibility of using this approach is related to the distance between the anterior maxillary wall and the anterior margin of the nasolacrimal duct. In their study, type I PLR (distance between 0–3 mm) was found in 31.5% of cases, type II (distance between 3–7 mm) was found in 56% of cases, and type III (distance higher than 7 mm) in only 12.5% of cases. In cases with type I and type II PLR, the PLRA is only possible with bone removal and lacrimal dislocation, while in type III, there is a need for little bone work without dislocation of the nasolacrimal duct. They also state that in these cases, the PLRA gives good direct visualization for the lateral pterygoid and infratemporal fossae.

In our study, we found a type I PLR in 22 cases (35%), a type II PLR in 31 cases (53.30%), and type III in 7 cases (11.66%). For type II and type III PLR, we continued our evaluation and measured the angles between the anterior maxillary wall and medial maxillary wall (angle 1) and the lateral margin of the nasolacrimal duct (angle 2) to see how much the angle is enclosed by the duct, and how direct visualization for the pterygopalatine fossa is obstructed in this type of approach.

We found that angle 1 had a mean value of 80.8° (minimum 75.5°, maximum 85.8°), while angle 2 had a mean value of 59.1° (minimum 57.6°, maximum 60.1°). The differences between the two angles were given by the width of the canal of the nasolacrimal duct, which encloses the angle of visualization at more than 30°. Considering this, the PLRA would be a very good tool for the anterior and inferior aspects of the maxillary sinus, as well as for the infratemporal fossa and more lateral aspect of the pterygopalatine fossa, but direct visualization of the medial part of the pterygopalatine fossa with a 0° telescope and instrumentation with straight instruments would be difficult through a PLRA without dislocating the nasolacrimal duct. In these cases, if PLRA is preferred, angled telescopes and instruments might be of better use. Also, in cases where better visualization is needed, combined approaches (through the middle meatus and pre-lacrimal fossa), medial maxillectomy or canine fossa approaches could offer better access to the whole pterygopalatine fossa. In a cadaveric dissection study, Cavallo et al. [11] concluded that the endoscopic endonasal approach was safe and effective for the removal of lesions in the pterygopalatine fossa, although they did not use a modified endonasal middle meatal transpalatine and endonasal middle meatal transantral approach for their dissections, and not a PLRA. Bing Zhou et al. [10] showed that the PLRA is safe and provides good access to the pterygopalatine and infratemporal fossae with preservation of lateral nasal wall structures and good surgical outcomes.

After analyzing our results, we consider that the angle of visualization should be taken into consideration in surgical planning when PLRA is proposed for lesions of the pterygopalatine fossa, especially for its medial part.

## CONCLUSIONS

The PLRA, when feasible (types II and III PLR), is a very good option for visualization and instrumentation of the anterior maxillary wall as well as for the alveolar recess and in reaching the infratemporal fossa and lateral part of the pterygopalatine fossa. In cases where lesions are situated in the medial part of the pterygopalatine fossa, measurements of the angles of access through the PLR should be considered in surgical planning.
